# Metagenomic Analysis of Gut Microbiota Structure and Function in Adults with Subclinical Hypothyroidism: A Cross-Sectional Study in China

**DOI:** 10.3390/microorganisms13112643

**Published:** 2025-11-20

**Authors:** Xueqing Li, Xue Ma, Lizhi Wu, Zhe Mo, Zhijian Chen, Ronghua Zhang, Mingluan Xing

**Affiliations:** Zhejiang Provincial Center for Disease Control and Prevention, 3399 Bin Sheng Road, Binjiang District, Hangzhou 310051, China; xqli@cdc.zj.cn (X.L.); xma@cdc.zj.cn (X.M.); lzhwu@cdc.zj.cn (L.W.); zhmo@cdc.zj.cn (Z.M.); zhjchen@cdc.zj.cn (Z.C.)

**Keywords:** subclinical hypothyroidism, gut microbiota, metagenomic analysis, thyroid function

## Abstract

Subclinical hypothyroidism (SCH) is a condition characterized by thyroid hormone dysregulation, often associated with subtle clinical symptoms and metabolic disturbances. Emerging evidence suggests that the gut microbiota plays a crucial role in modulating thyroid function, but the microbiota–thyroid axis in SCH remains poorly understood. This study systematically investigates the gut microbiota composition, functional characteristics, and their correlation with thyroid hormone profiles in SCH patients. Using metagenomic sequencing and thyroid function assessments, we identified significant alterations in the gut microbiota of SCH patients, including a depletion of beneficial microbes such as *Blautia* and *Bifidobacterium*, and an enrichment of opportunistic pathogens like *Bacteroides* and *Escherichia*. Notably, *Blautia* depletion was negatively correlated with TSH levels, while *Bacteroides* abundance positively correlated with TSH levels, further highlighting the role of gut microbiota in thyroid dysfunction. Moreover, functional gene analysis revealed significant alterations in microbial metabolic pathways, with key pathways demonstrating correlations with thyroid hormone levels (free triiodothyronine (FT3) and triiodothyronine (T3)). Our findings suggest that gut microbial dysbiosis is closely associated with SCH. The study provides novel insights into the gut–thyroid axis and its role in SCH, offering new targets for early diagnosis, risk stratification, and intervention strategies in thyroid diseases.

## 1. Introduction

Hypothyroidism, a prevalent endocrine disorder, is characterized by an insufficient secretion of thyroid hormones, unable to meet the peripheral tissue demands, and typically necessitates lifelong replacement therapy [1,2]. Based on severity, hypothyroidism is classified into overt hypothyroidism (OH) and subclinical hypothyroidism (SCH). Furthermore, autoimmune thyroid diseases, such as Hashimoto’s thyroiditis and Graves’ disease, represent a major etiology of thyroid dysfunction [3,4]. Hashimoto’s thyroiditis can cause chronic inflammation of the thyroid tissue, and approximately 20% to 30% of patients may develop symptoms of hypothyroidism, while Graves’ disease is an autoimmune disorder typically resulting in hyperthyroidism [4]. SCH is commonly regarded as an early stage of thyroid dysfunction, marked by elevated serum thyroid-stimulating hormone (TSH) levels while free thyroxine (FT4) levels remain within normal ranges [5]. With the advancement of modernity, SCH has become increasingly prevalent, affecting up to 10% of the adult population [6]. While most individuals with SCH remain asymptomatic, approximately 2–5% of SCH patients annually progress to OH [7], emphasizing the importance of early identification of high-risk individuals. Moreover, hypothyroidism has been associated with cardiovascular dysfunction and is recognized as a risk factor for heart failure [8]. It also impacts the neurological, musculoskeletal, and gastrointestinal systems to varying degrees [9], which is associated with a myriad of health complications, including cardiovascular diseases, infertility, and impaired cerebral development in children [10,11,12]. Hence, a deeper investigation into SCH is crucial for elucidating its pathophysiological mechanisms and preventing its progression to OH.

The etiologies of thyroid diseases are highly diverse and complex, involving both genetic susceptibility and environmental factors [13]. Previous research has predominantly focused on the relationship between thyroid diseases and common environmental influences, such as lifestyle factors. However, in recent years, the gut microbiota, as a critical regulator of host health, has garnered significant attention. It impacts host physiology through mechanisms such as energy homeostasis regulation, vitamin synthesis, immune modulation, and maintenance of the intestinal barrier [14,15,16]. Prior studies have established links between gut microbiota and various thyroid disorders, including Graves’ disease, Hashimoto’s thyroiditis, and thyroid cancer, leading to the conceptualization of the “gut–thyroid axis” [17,18]. While the importance of the gut microbiota in human health is well-documented, research concerning its role in thyroid function, particularly in SCH, remains limited. Thus, the relationship between the gut microbiota and SCH warrants further investigation to enhance understanding of the “gut–thyroid axis.”

Although the impact of hypothyroidism on the diversity and composition of the human gut microbiota has been established in prior studies, its effects on the functional aspects of the microbiota remain poorly understood. To address this gap, the present study proposes an initial exploration aimed at elucidating the relationship between gut microbiota functionality and SCH. Participants were conveniently recruited from health centers across three counties in Zhejiang Province, China, comprising adults aged 20–71 years, in order to capture a broad yet metabolically stable demographic and avoid confounding effects related to puberty, aging, or age-related diseases. Fecal samples were subjected to metagenomic sequencing, and serum samples were analyzed for thyroid function to investigate the gut microbiota functionality in SCH patients. The results were compared with those from healthy controls and OH patients, thereby aiming to explore the relationship between SCH and the gut microbiota in adults.

## 2. Materials and Methods

### 2.1. Study Population

A cross-sectional study was conducted to examine the gut microbiota in subclinical hypothyroidism patients. The study subjects were adults aged 20 to 71 years from three counties in Zhejiang Province, China. The exclusion criteria were as follows: (1) individuals with psychiatric disorders or cognitive impairments (e.g., dementia, comprehension deficits, deaf-mutism); (2) newly diagnosed or currently treated cancer patients; (3) gastrointestinal diseases or infections; (4) recent (<3 months prior) use of iodinated contrast agents or amiodarone; (5) no antibiotics, antibacterial agents, or thyroid medications were administered to the recruited subjects in the previous three months. A total of 277 adults were included in this preliminary study, and their characteristics are presented in Table 1. Referencing Professor Teng’s research [19] and combined with the reference range of our research laboratory. The euthyroidism reference range was defined as TSH within 0.27–4.2 mIU/L and FT4 within 12–22 pmol/L, SCH was diagnosed as TSH > 4.2 mIU/L and FT4 within 12–22 pmol/L, and OH was diagnosed as TSH > 4.2 mIU/L and FT4 < 12 pmol/L. The study protocol was approved by the Zhejiang Provincial Center for Disease Control and Prevention (Approval No: 2020–040–01). All participants were informed of the nature of the study and were required to provide written informed consent.

### 2.2. Data Collection

All participants underwent standardized questionnaire surveys and physical examinations conducted by trained and evaluated professional technicians. Anthropometric measurements, such as height and weight, were collected by trained health technicians. Body mass index (BMI) was calculated as body mass (kg) divided by height squared (m^2^).

On the day of the physical examinations, fasting blood and fecal samples were collected at the local health clinic. Serum samples were collected via venipuncture into 5 mL Vacuette tubes containing procoagulant. Thirty minutes after collection, the samples were centrifuged at 3000 rpm for 10 min. Samples were shipped the same day via a cold chain logistics system to the central laboratory in Hangzhou, China, for immediate centralized testing. For fecal sample collection, participants were provided with sterile fecal collection containers and given clear instructions to avoid contamination with urine, water, or toilet paper. Fresh fecal samples (5–10 g) were collected directly into the container or transferred using a clean liner. The samples were immediately sealed and labeled with the participant’s ID, collection date, and time. Samples were stored at 4 °C and transported to the research facility within 24 h, frozen at −80 °C for long-term preservation.

### 2.3. Thyroid Function Test

Serum levels of TSH, free triiodothyronine (FT3), triiodothyronine (T3), FT4, thyroxine (T4) and thyroglobulin (Tg) were measured using the Cobas e601 analyzer (Roche Diagnostics GmbH, Mannheim, Germany) employing electrochemiluminescence immunoassays, in combination with the appropriate calibration materials, reagents, and quality controls. Quality control procedures were implemented strictly according to the manufacturer’s instructions before, during, and after testing. The coefficients of variation (CVs) for the control samples (PreciControl Universal & PreciControl ThyroAB, Roche, Germany) were as follows: TSH 2.75–5.57%, FT3 3.44–5.26%, T3 3.55–5.03%, FT4 2.26–4.23%, T4 4.50–5.17%, and Tg 3.01–4.27%.

### 2.4. Shotgun Metagenomic Sequencing

Total stool genomic DNA was extracted from both stools with the QIAamp DNA Stool Mini Kit (Qiagen, Hilden, Germany) according to the manufacturer’s instructions. The subsequent library construction and sequencing process was commissioned to Majorbio Bio-Pharm Technology Co., Ltd. (Shanghai, China). Briefly, the DNA extract was fragmented to an average size of about 300 bp for paired-end library construction. The paired-end library was constructed using NEXTFLE Rapid DNA-Seq. Paired-end sequencing was performed on Illumina Novaseq X plus (Illumina Inc., San Diego, CA, USA) according to the manufacturer’s instructions. Metagenome sequencing data was further processed by bioinformatics pipelines on the online platform of Majorbio Cloud Platform (www.majorbio.com (accessed on 26 May 2025)) with data quality-filtering (length < 50 bp or with a quality value < 20 or having N bases), assembly, genomic contamination elimination and taxonomic compositions identification. Details of the processing, taxonomic classification, and functional annotation of the metagenomic sequencing data are provided in the Supplementary Methods.

### 2.5. Statistical Analyses

The Kolmogorov–Smirnov method was adopted to test the normality of continuous variable distributions. Normally distributed data were expressed as mean ± standard deviation (SD) and a *t*-test for comparison between groups. Non-normally distributed data were expressed as the median with the interquartile range (IQR: 25th–75th percentiles) and analyzed using the Wilcoxon rank-sum test for comparison of two groups. Categorical variables were represented by counts and percentages, with the chi-squared (χ^2^) test or Fisher’s exact test employed for comparisons between groups. The Ace, Sobs, Chao, Shannon and Simpson indices were used for alpha diversity analysis with the Kruskal–Wallis test to examine the significance among different groups. The similarity among the microbial communities in different samples was determined by principal coordinate analysis (PCoA) based on Bray–Curtis distance. Permutational multivariate ANOVA (PERMANOVA) was used to examine the significance of differences in microbial community structure between groups. The PCoA was performed based on untransformed PRKM quantitative data, using the Bray–Curtis distance algorithm. Differences in species-level similarity based on Bray–Curtis distance between samples across different groups were assessed using the Kruskal–Wallis test. The linear discriminant analysis (LDA) and effect size (LEfSe) analysis [20] were performed to identify the significantly abundant taxa (phylum to genera) of bacteria among the different groups (LDA score > 2.5, *p* < 0.05). For all high-dimensional analyses, including differential abundance testing of taxa and functional pathways, as well as correlation matrices, *p*-values were adjusted for multiple testing using the Benjamini–Hochberg False Discovery Rate (FDR) method. Spearman correlation was used to analyze the correlation between thyroid function and both microbial species and microbial pathways. To identify multivariate associations between microbial species and KEGG Orthology (KO) terms versus thyroid function indices, the same covariate adjustments (age, sex, BMI) were applied consistently to both the taxonomic and functional analyses using Multivariate Association with Linear Models (MaAsLin2, version 1.7.3, accessed on 2 November 2021). The significance of associations was determined based on an FDR-corrected q-value threshold of <0.05. All tests were two-tailed, and significance was set at a 0.05 level (*p* < 0.05).

## 3. Results

### 3.1. Participant Characteristics

A total of 277 participants were included in this study, comprising 50 individuals with SCH, 5 individuals with OH, and 222 euthyroidism controls. As presented in Table 1, the mean ± SD age and BMI of the participants were 49.66 ± 12.30 years and 24.48 ± 3.88 kg/m^2^, respectively, with the majority (64.62%) being female. No statistically significant differences were observed in age, sex, or BMI among the three groups.

The concentrations of FT3, T3, FT4, T4, TSH, and Tg were measured as follows: 4.98 (4.54, 5.42) pmol/L, 1.91 (1.72, 2.16) nmol/L, 16.11 (14.90, 17.71) pmol/L, 116.10 (100.70, 130.20) nmol/L, 2.41 (1.69, 3.65) mIU/L, and 11.38 (6.52, 19.05) ng/mL, respectively. Compared to the euthyroidism group, both the SCH (*p* < 0.001) and OH (*p* < 0.001) groups exhibited significantly lower FT4 levels. The OH group additionally displayed decreased T4 levels (*p* = 0.003). Conversely, TSH levels were significantly higher in both the SCH (*p* < 0.001) and OH (*p* < 0.001) groups relative to controls, while Tg levels were notably elevated in the SCH group (*p* = 0.001).

### 3.2. Gut Microbiota Diversity Analysis

**Figure 1:**
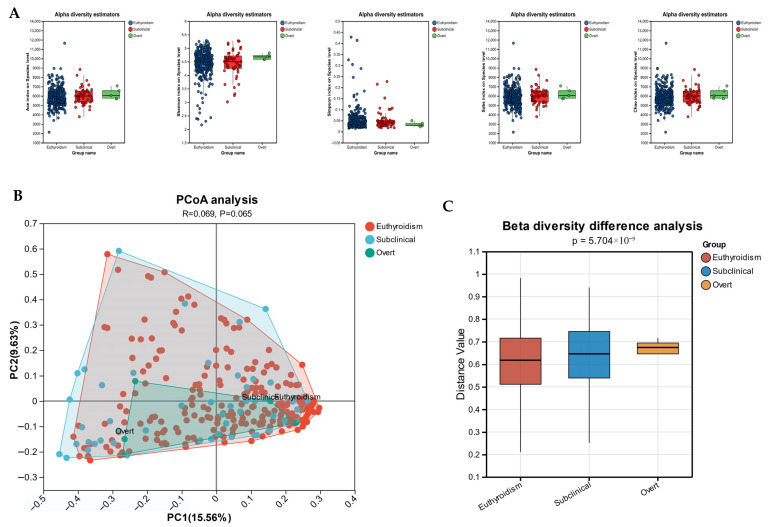
Comparison of alpha and beta diversity of gut microbiota among euthyroidism (n = 222), Subclinical Hypothyroidism (SCH, n = 50), and Overt Hypothyroidism (OH, n = 5) groups. (**A**) Alpha diversity analysis assessed by the Ace, Shannon, Simpson, Sobs, and Chao indices. The center line in the boxplots represents the median and the box limits indicate the interquartile range (IQR). (**B**) Principal component (PCoA) analysis based on Bray–Curtis distance. Permutational multivariate ANOVA (PERMANOVA) was used to examine the significance of differences in microbial community structure among groups (R = 0.069, *p* = 0.065). X- and Y-axes represent the first PCoA1 and the second PCoA2, respectively. The percentage in the brackets represents the relative contribution of the component to the total difference. Each sample corresponded to one dot in the graph. Different groups are represented by different colors. (**C**) Differences in species-level similarity based on Bray–Curtis distance between samples across different groups were assessed using the Kruskal–Wallis test (*p* < 0.001).

We compared the gut microbiota diversity among the different groups using α-diversity and β-diversity analyses (Figure 1). In the α-diversity analysis, the Ace, Sobs and Chao indices were used to estimate species richness, while the Shannon and Simpson indices assessed microbiota richness and evenness. No significant differences in these indices were observed among the three groups (Figure 1A). To assess β-diversity, the overall microbial community structure was compared using PCoA based on Bray–Curtis distances. While the PCoA plot revealed substantial overlap among the Euthyroidism, SCH, and OH groups with no clear clustering (Figure 1B; PERMANOVA: R = 0.069, *p* = 0.065), a subsequent analysis of inter-sample similarities showed a statistically significant difference in β-diversity (Kruskal–Wallis test: *p* < 0.001; Figure 1C). This suggests that although the groups’ microbial communities are not distinctly separated in the reduced dimensional space, their compositional structures are significantly different in terms of overall similarity.

### 3.3. Characteristic Gut Microbiota Alterations

**Figure 2:**
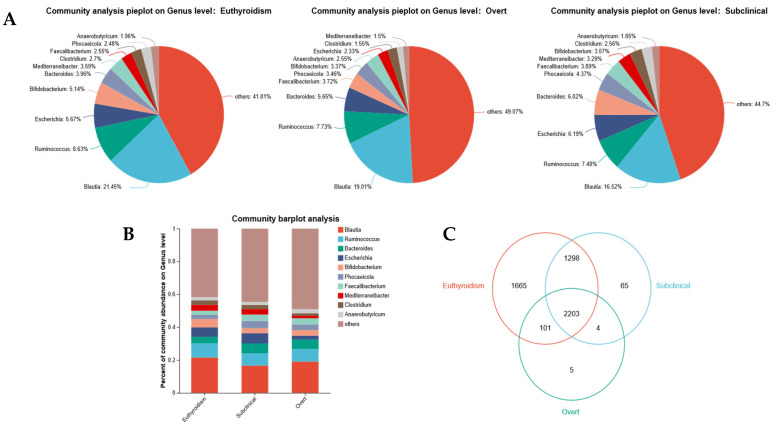
Composition of gut microbiota at the genus level among the three groups. (**A**) Community pie charts showing the relative abundance of the top 10 genera in each group. (**B**) Community bar plot analysis displaying the relative abundance of bacterial genera across all samples. (**C**) Venn diagram illustrating the number of genera shared among the Euthyroidism, SCH, and OH groups, as well as those unique to each group, under the current sequencing depth and detection threshold.

At the genus level, we analyzed the composition and relative abundance of the gut microbiota (Figure 2A,B). The genera *Blautia* and *Ruminococcus* exhibited the highest relative abundances, followed by *Escherichia*, *Bifidobacterium*, *Bacteroides*, and *Faecalibacterium*. Compared to the euthyroidism group, both the SCH and OH groups showed decreased relative abundances of *Blautia*, *Ruminococcus*, *Bifidobacterium*, *Mediterraneibacter*, and *Clostridium*, while the relative abundances of *Bacteroides*, *Phocaeicola*, and *Faecalibacterium* increased. The relative abundance of *Escherichia* was highest in the SCH group and lowest in the OH group. Under the current sequencing depth and detection threshold, we compared the distribution of bacterial genera across the three groups (Figure 2C). The analysis revealed 4 genera that were shared between the SCH and OH groups but were not detected in the euthyroidism group. Additionally, 65 genera were identified in the SCH group, while the OH group had 5 unique genera (Table S1). It is important to note that these ‘unique genera’ may reflect detection thresholds and sampling variance, and their biological relevance requires further investigation.

**Figure 3:**
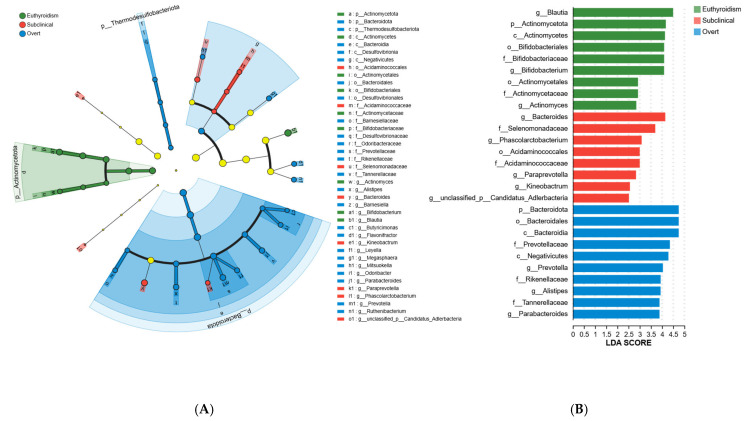
The differential microbiota at various taxonomic levels between the three groups was determined by linear discriminant analysis (LDA) and effect size (LEfSe) analysis (LDA value > 2.5, *p* < 0.05). (**A**) LEfSe taxonomic cladogram. (**B**) Histogram of the LDA scores. The letter before the name of the bacteria indicates different taxa levels. g, indicates genus; f, indicates family; o, indicates order; c, indicates class; p, indicates phylum.

To further identify key microbial groups, we performed a LEfSe test to compare the gut microbiota at various taxonomic levels. As shown in Figure 3, we selected taxa with an LDA score greater than 2.5 for comparison, identifying 27 microbial groups with significant inter-group differences. The characteristic gut microbiota of the SCH group included *Bacteroides*, Selenomonadaceae, *Phascolarctobacterium*, Acidaminococcales, Acidaminococcaceae, *Paraprevotella*, *Kineobactrum*, and the unclassified genus Candidatus Adlerbacteria (Figure 3B). Notably, *Phascolarctobacterium* belongs to the Acidaminococcaceae family, Acidaminococcales order, Negativicutes class; Selenomonadaceae belongs to the Negativicutes class; *Paraprevotella* belongs to the Prevotellaceae family, Bacteroidales order, Bacteroidia class, Bacteroidota phylum; and *Bacteroides* belongs to the Bacteroidales order, Bacteroidia class, Bacteroidota phylum (Figure 3A). The characteristic microbiota of the OH group included Bacteroidota, Bacteroidales, Bacteroidia, Prevotellaceae, Negativicutes, *Prevotella*, Rikenellaceae, *Alistipes*, Tannerellaceae, and *Parabacteroides*. In contrast, the euthyroidism group’s characteristic microbiota included *Blautia*, Actinomycetota, Actinomycetes, Bifidobacteriales, *Bifidobacteriaceae*, Bifidobacterium, Actinomycetales, Actinomycetaceae, and *Actinomyces*.

### 3.4. Correlation Analysis Between Thyroid Function Indicators and Gut Microbiota

**Figure 4:**
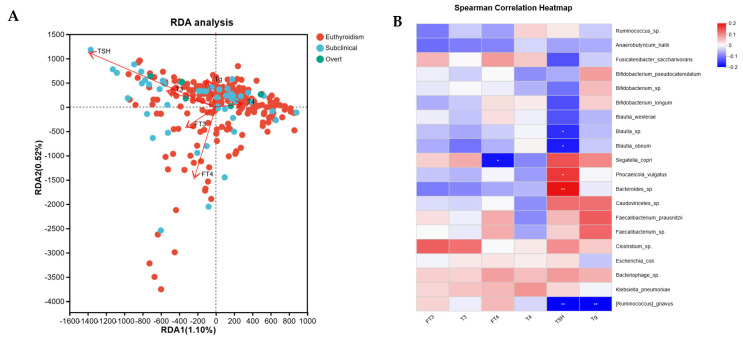
Association between gut microbiota and thyroid function parameters. (**A**) Redundancy analysis (RDA) was used to evaluate the possible association of gut microbes with thyroid function parameters. The angle between any two arrows is representative of the correlation between species and thyroid function parameters. The acute angle indicates positive correlation, and the obtuse angle indicates a negative correlation. (**B**) Spearman correlation between thyroid function parameters and the top 20 abundant gut microbial species. Correlation coefficients are color-coded, with asterisks indicating statistical significance. Red represents a positive correlation, and blue represents a negative correlation. *, *p* < 0.05; **, *p* < 0.01.

To explore the relationship between the gut microbiota and thyroid function, we conducted redundancy analysis (RDA) and Spearman correlation analysis to examine the association between the relative abundance of microbiota at the genus level and six thyroid function indicators (Figure 4). In the RDA, longer arrows indicate a stronger correlation between thyroid function indicators and microbiota distribution and composition. Acute angles between arrows suggest positive correlations, while obtuse angles indicate negative correlations. The results showed that TSH and FT4 exhibited the strongest correlation with gut microbiota distribution. TSH was positively correlated with T3 and FT3, and negatively correlated with T4 (Figure 4A). The relative abundance of *Blautia*_sp., *Blautia_obeum*, and *[Ruminococcus]_gnavus* was negatively correlated with TSH, whereas *Phocaeicola_vulgatus* and *Bacteroides*_sp. were positively correlated with TSH. Additionally, the relative abundance of *Segatella_copri* was negatively correlated with FT4, and *[Ruminococcus]_gnavus* was negatively correlated with Tg (Figure 4B).

### 3.5. Functional Gene Analysis of the Gut Microbiota

**Figure 5:**
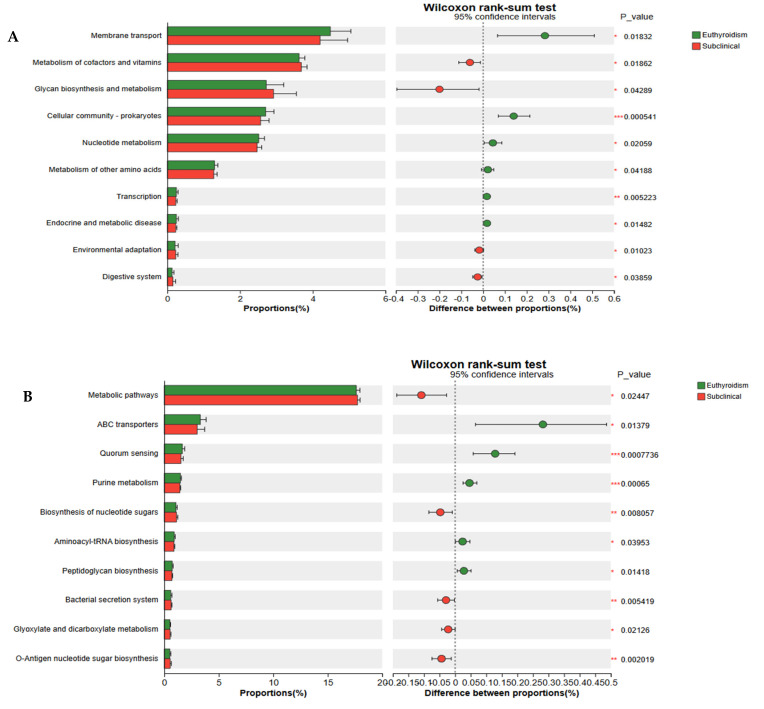
Differential abundance of predicted microbial metabolic pathways between the SCH and euthyroidism groups, based on the Kyoto Encyclopedia of Genes and Genomes (KEGG). (**A**) Differential functions in KEGG level 2 between SCH and euthyroidism groups; (**B**) Differential functions in KEGG level 3 between SCH and euthyroidism groups. The Wilcoxon rank-sum test was used to detect significant changes. *, *p* < 0.05; **, *p* < 0.01; ***, *p* < 0.001.

To better understand the metabolic functional changes in the gut microbiota, we analyzed the functional genes using the Kyoto Encyclopedia of Genes and Genomes (KEGG) database and applied the Wilcoxon rank-sum test to compare functional gene abundances between the groups (Figure 5). At level 2 (Figure 5A), the gut microbiota of the SCH group exhibited significantly higher gene abundances in pathways related to metabolism of cofactors and vitamins, glycan biosynthesis and metabolism, environmental adaptation, and digestive systems compared to the euthyroidism group. Conversely, pathways associated with membrane transport, cellular community-prokaryotes, nucleotide metabolism, metabolism of other amino acids, transcription, and endocrine and metabolic diseases were significantly lower in the SCH group. At level 3 (Figure 5B), significant increases in pathway gene abundances in the SCH group were observed in metabolic pathways, biosynthesis of nucleotide sugars, bacterial secretion systems, glyoxylate and dicarboxylate metabolism, and O-antigen nucleotide sugar biosynthesis. Pathways such as ATP-binding cassette (ABC) transporters, quorum sensing, purine metabolism, aminoacyl-tRNA biosynthesis, and peptidoglycan biosynthesis were significantly lower in the SCH group. Additionally, the metabolic pathway gene abundances between the euthyroidism and OH groups were compared, with the euthyroidism group showing significantly lower abundances in replication and repair pathways (level 2). Notable differences at level 3 were observed in several pathways, including glycolysis/gluconeogenesis, homologous recombination, and pentose phosphate pathways (Figure S1).

**Figure 6:**
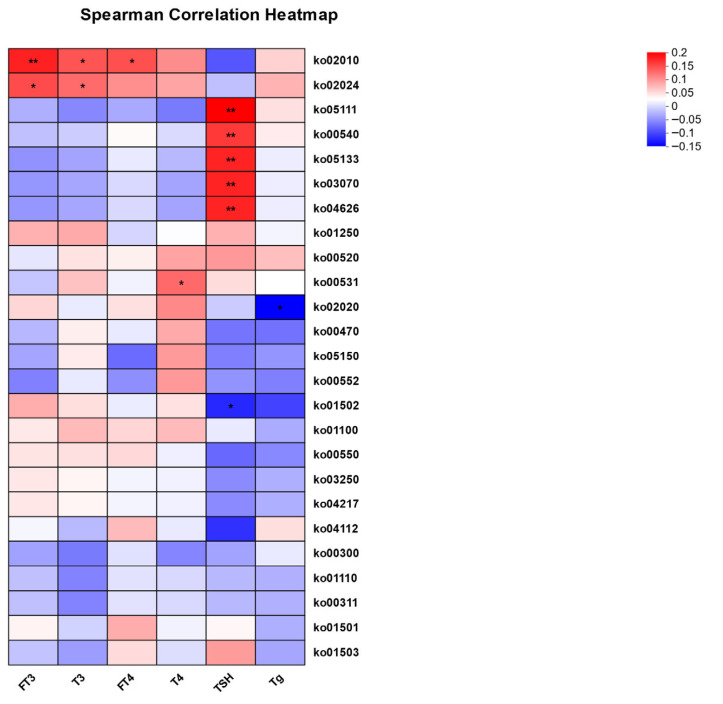
Correlation analysis between thyroid function parameters and the relative abundance of specific KEGG Orthology (KO) pathways. The heatmap displays Spearman correlation coefficients, with asterisks indicating statistical significance. Red represents a positive correlation, and blue represents a negative correlation. *, *p* < 0.05; **, *p* < 0.01.

A specific focus was placed on the relationship between microbial pathways and thyroid function. Based on the KEGG database, correlation coefficients for microbial functional units (KO pathways) and thyroid function indicators were computed, with a heatmap of these correlations presented (Figure 6). The results indicated positive correlations between KO02010 with FT3, T3, and FT4, as well as KO02024 with FT3 and T3. Furthermore, KO05111, KO00540, KO05133, KO03070, and KO04626 showed positive correlations with TSH.

To further investigate the multivariate associations between gut microbial features and thyroid function, we performed a MaAsLin2 analysis of microbial species and KO terms against thyroid indices, adjusting for sex, age, and BMI. This analysis revealed 460 species and 68 KO terms that were significantly associated with thyroid function parameters (q-value < 0.05; Tables S2 and S3). These statistically robust associations highlight specific microbial functions implicated in thyroid physiology, thereby pinpointing key functional features for targeted future research on the gut–thyroid axis.

## 4. Discussion

This study systematically elucidates the functional characteristics of the gut microbiota in SCH patients and its association with thyroid hormone dysregulation, further advancing the understanding of the “gut–thyroid axis” mechanism. The results reveal significant alterations in the gut microbiota of SCH patients, characterized by a depletion of beneficial commensals such as *Blautia* and *Bifidobacterium*, alongside an enrichment of opportunistic pathogens like *Bacteroides* and *Escherichia*. Additionally, the gut microbiota in SCH patients exhibited patterns consistent with metabolic imbalance and dysregulation of quorum-sensing pathways. Furthermore, correlations between specific microbial taxa and stress-response and transport-related pathways with thyroid function indicators substantiate the pivotal role of the gut–thyroid axis. This study provides new targets for the early diagnosis, risk stratification, and intervention of SCH, and offers a novel perspective on microbial regulation in the treatment of thyroid diseases, holding significant clinical translational value.

Through metagenomic sequencing and thyroid function assessment, we comprehensively identified the abnormal characteristics of the gut microbiota in SCH patients and performed comparative analyses with healthy controls. The microbiota of SCH patients exhibited a distinctive dysbiosis, primarily marked by the depletion of beneficial microbiota such as *Blautia* and *Bifidobacterium*, and the overgrowth of opportunistic pathogens like *Bacteroides* and *Escherichia*. Previous studies have similarly found a significant reduction in *Blautia* in patients with hypothyroidism and Graves’ disease, aligning with our findings [21]. *Blautia* is a key producer of butyrate, a metabolite known to play an anti-inflammatory role by enhancing the intestinal mucosal barrier and maintaining epithelial integrity [22]. Research by Nagendra Singh et al. has shown that butyrate activates the G-protein-coupled receptor 109a signaling pathway, promoting the production of regulatory T cells and interleukin-10, inducing T-cell proliferation and enhancing interleukin-8 secretion, thus exerting systemic anti-inflammatory effects [23]. The negative correlation between *Blautia* and TSH further supports a potential link between its depletion and elevated TSH levels, which serves as a core diagnostic marker of SCH.

In parallel, the reduction in *Bifidobacterium* is commonly observed in both hypothyroidism and hyperthyroidism, as supported by prior research [18]. Notably, *Bifidobacterium* is positively correlated with the absorption of trace elements such as selenium and zinc, and its depletion could potentially reduce the bioavailability of these elements in the host. Selenium, as a critical cofactor for deiodinases, plays a direct role in the conversion of T4 to T3 [24]. Hence, the gut microbiota may modulate thyroid function by influencing the uptake of thyroid-related micronutrients [25]. In contrast, the proliferation of opportunistic pathogens further aggravates thyroid dysfunction. Based on previous research [24], lipopolysaccharides (LPS) are known to interfere with the expression and activity of the sodium-iodide symporter (NIS), thereby affecting iodine uptake and thyroid hormone synthesis. This established mechanism provides a plausible explanation for how the observed enrichment of Escherichia might be linked to thyroid dysfunction. *Escherichia* overgrowth releases lipopolysaccharides (LPS), which interfere with the expression and activity of the sodium-iodide symporter (NIS), thereby affecting iodine uptake and thyroid hormone synthesis [26]. In agreement with previous findings, Ishaq et al. observed excessive growth of *Bacteroides* in hypothyroid Hashimoto’s thyroiditis patients, and Jiang et al. reported significantly higher *Bacteroides* abundance in patients with Graves’ disease compared to controls, aligning with our results [27,28]. *Bacteroides* produces short-chain fatty acids (SCFAs) such as succinate, propionate, and acetate, according to previous studies, which do not induce mucin synthesis [28,29]. The gut microbiota may also affect thyroid function by producing SCFAs that reduce gut pH and enhance the bioavailability of colonic iron. Notably, we found a significant positive correlation between *Bacteroides* abundance and TSH levels, as TSH exceeds the normal threshold in SCH, suggesting that *Bacteroides* enrichment could serve as a potential microbial biomarker for auxiliary diagnosis of SCH. The characteristic dysbiosis of the gut microbiota not only provides new microbiological insights into SCH but also offers potential intervention strategies for its prevention and management. Probiotic supplementation with *Blautia* and *Bifidobacterium*, alongside inhibition of excessive proliferation of *Escherichia* and *Bacteroides*, may serve as effective therapeutic avenues.

Additionally, functional gene analysis of the gut microbiota revealed significant associations between microbial dysbiosis and SCH, which is consistent with the idea that gut microbiota dysfunction may influence thyroid hormone homeostasis through multiple mechanisms. The study demonstrated alterations in pathways related to the metabolism of auxiliary factors and vitamins, membrane transport, and ABC transporters. Specifically, the KO02010 (ABC transporters) and KO02024 (Quorum sensing) pathways exhibited positive correlations with FT3 and T3, suggesting that they may be linked to thyroid hormone synthesis by modulating the intestinal absorption of micronutrients such as iodine. This finding aligns with research by Vought et al., which demonstrated that the gut microbiome depleted by antibiotic treatment reduces thyroid iodine uptake in rats [30]. Furthermore, lipopolysaccharide (LPS) biosynthesis (KO00540) was positively correlated with TSH levels, indicating that gut microbiota may interfere with thyroid function via LPS. Previous studies have shown that LPS can stimulate the expression of thyroglobulin (Tg) and NIS genes in thyroid cells through the activation of TSH receptors, as well as activate nuclear factor kappa-B (NF-kB) signaling through Toll-like receptor 4 (TLR4), modulating thyroid cell inflammation [31,32]. On the other hand, LPS activates thyroid cell inflammation via the NF-κB signaling pathway mediated by TLR4 [33]. Collectively, these findings imply that specific functional pathways of the gut microbiota may be involved in SCH by affecting nutrient absorption and inflammatory processes, offering new insights for microbiota-oriented approaches in thyroid disorders.

However, some limitations still existed in the present study. First, due to the low prevalence of overt hypothyroidism, the sample size of the OH group in this study was relatively small (n = 5), which may limit the statistical power and generalizability of the findings related to this specific patient group. To mitigate this potential bias, we performed a supplementary analysis comparing the gut microbiota composition between the euthyroidism and SCH groups. The results of this analysis, which are consistent with our primary conclusions, are provided in the Supplementary Materials (Figures S2 and S3). Large-scale studies with sufficient statistical power are still needed to further confirm the impact of gut microbiota alterations on overt hypothyroidism. Second, the participants covered a wide age range (20–71 years), and gut microbiota composition may change with age. Although no significant age difference was found among groups in our study, future studies with larger sample sizes stratified by age groups (e.g., young adults, middle-aged, and elderly) are warranted to confirm our findings. Third, the lack of adjustment for potential confounders such as detailed dietary habits and thyroid autoantibody status in our differential abundance and correlation analyses represents a limitation. This limitation precludes us from determining whether the observed microbial alterations are specifically associated with autoimmune thyroiditis or other etiologies, and we could not control for the potential confounding effects of iodine deficiency or excess. Future studies with larger cohorts and more comprehensive covariate data are warranted to confirm these associations.

## 5. Conclusions

In conclusion, our study reveals a link between gut microbial dysbiosis and SCH. The depletion of beneficial microbes and the enrichment of opportunistic pathogens in SCH patients were associated with thyroid hormone imbalances. These findings emphasize the gut–thyroid axis as a potential therapeutic target for microbiota-based interventions in managing thyroid dysfunction, offering new opportunities for early diagnosis and treatment strategies in thyroid diseases.

## Figures and Tables

**Table 1:** Characteristics of the study population (n = 277).

Characteristics	Total (n = 277)	Euthyroidism (n = 222)	SCH (n = 50)	OH (n = 5)	*p* ^a^
Age, years, Mean ± SD	49.66 ± 12.30	49.08 ± 12.26	51.82 ± 12.29	53.80 ± 13.37	0.272
BMI, kg/m^2^, Mean ± SD	24.48 ± 3.88	24.31 ± 3.94	25.31 ± 3.69	23.60 ± 2.09	0.230
Sex, n (%)					0.059
Male	98 (35.38)	85 (38.29)	13 (26.00)	0 (0.00)	
Female	179 (64.62)	137 (61.71)	37 (74.00)	5 (100.00)	
FT3, pmol/L, M (Q_1_, Q_3_)	4.98 (4.54, 5.42)	5.04 (4.55, 5.42)	4.75 (4.54, 5.43)	4.35 (3.78, 4.92)	0.078
T3, nmol/L, M (Q_1_, Q_3_)	1.91 (1.72, 2.16)	1.92 (1.72, 2.16)	1.92 (1.70, 2.21)	1.78 (1.71, 1.91)	0.732
FT4, pmol/L, M (Q_1_, Q_3_)	16.11 (14.90, 17.71)	16.47 (15.20, 17.98)	15.04 (13.75, 16.45) ^#^	11.24 (10.58, 11.71) ^#^	<0.001
T4, nmol/L, M (Q_1_, Q_3_)	116.10 (100.70, 130.20)	117.20 (102.50, 130.65)	110.00 (97.85, 127.93)	83.01 (82.90, 91.25) ^#^	0.009
TSH, µIU/mL, M (Q_1_, Q_3_)	2.41 (1.69, 3.65)	2.00 (1.50, 2.89)	5.13 (4.57, 5.96) ^#^	7.57 (5.27, 8.76) ^#^	<0.001
Tg, ng/mL, M (Q_1_, Q_3_)	11.38 (6.52, 19.05)	10.10 (5.99, 17.41)	16.32 (11.88, 21.97) ^#^	7.43 (0.15, 16.16)	<0.001

^a^ Chi-square tests for nominal variables, an ANOVA for continuous variables, and a Kruskal–Wallis test to detect the difference in thyroid function levels between the three groups. Post hoc pairwise comparisons were conducted using Dunn’s procedure with Bonferroni correction. ^#^ Compared with the euthyroidism group, the difference was statistically significant. Abbreviations: BMI, body mass index; SD, standard deviation; M, median; FT3, free triiodothyronine; T3, triiodothyronine; FT4, free thyroxine; T4, thyroxine; TSH, thyroid-stimulating hormone; Tg, thyroglobulin.

## Data Availability

The original contributions presented in this study are included in the article/Supplementary Materials. Further inquiries can be directed to the corresponding authors.

## Supplementary Materials

The following supporting information can be downloaded at: https://www.mdpi.com/article/10.3390/microorganisms13112643/s1, Supplementary Methods: Shotgun Metagenomic Sequencing; Figure S1: Differential functional analysis of the gut microbiota based on KEGG. (A) Differential functions in KEGG level 2 between OH and euthyroidism groups; (B) Differential functions in KEGG level 3 between OH and euthyroidism groups. The Wilcoxon rank-sum test was used to detect significant changes. *, *p* < 0.05; **, *p* < 0.01; Figure S2: Comparison of alpha and beta diversity of gut microbiota between euthyroidism (n = 222) and SCH (n = 50) groups. (A) Alpha diversity analysis assessed by the Ace, Shannon, Simpson, Sobs, and Chao indices. The center line in the boxplots represents the median and the box limits indicate the interquartile range (IQR). (B) Principal component (PCoA) analysis based on Bray-Curtis distance. Permutational multivariate ANOVA (PERMANOVA) was used to examine the significance of differences in microbial community structure between groups (R^2^ = 0.01, *p* = 0.002). X- and Y-axes represent the first PCoA1 and the second PCoA2, respectively. The percentage in the brackets represents the relative contribution of the component to the total difference. Each sample corresponded to one dot in the graph. Different groups are represented by different colors. (C) Differences in species-level similarity based on Bray-Curtis distance between samples across different groups were assessed using the Willcoxon test. ***, *p* < 0.001; Figure S3: The differential microbiota at various taxonomic levels between two groups was determined by linear discriminant analysis (LDA) and effect size (LEfSe) analysis (LDA value > 3, *p* < 0.05). (A) LEfSe taxonomic cladogram. (B) Histogram of the LDA scores. The letter in the former of the name of bacteria indicates different taxa levels. g, indicates genus; f, indicates family; o, indicates order; c, indicates class; p, indicates phylum; Table S1: Shared and Unique Bacterial Genera between Subclinical Hypothyroidism and Overt Hypothyroidism Groups; Table S2: Covariate-Adjusted Significant Associations between Gut Microbial KEGG Orthology and Thyroid Function Indices; Table S3: Covariate-Adjusted Significant Associations between Gut Microbial Species and Thyroid Function Indices.

## References

[1] Burekovic A., Halilovic D., Sahbaz A. (2022). Hypothyroidism and Subclinical Hypothyroidism as a Consequence of COVID-19 Infection. Med. Arch..

[2] McDermott M.T. (2020). Hypothyroidism. Ann. Intern. Med..

[3] Ragusa F., Fallahi P., Elia G., Gonnella D., Paparo S.R., Giusti C., Churilov L.P., Ferrari S.M., Antonelli A. (2019). Hashimotos’ thyroiditis: Epidemiology, pathogenesis, clinic and therapy. Best Pract. Res. Clin. Endocrinol. Metab..

[4] Smith T.J., Hegedüs L. (2016). Graves’ Disease. N. Engl. J. Med..

[5] LeFevre M.L. (2015). Screening for thyroid dysfunction: U.S. Preventive Services Task Force recommendation statement. Ann. Intern. Med..

[6] Biondi B., Cappola A.R., Cooper D.S. (2019). Subclinical Hypothyroidism: A Review. JAMA.

[7] Khandelwal D., Tandon N. (2012). Overt and subclinical hypothyroidism: Who to treat and how. Drugs.

[8] Abdel-Moneim AGaber A.M., Gouda S., Osama A., Othman S.I., Allam G. (2020). Relationship of thyroid dysfunction with cardiovascular diseases: Updated review on heart failure progression. Hormones.

[9] Chaker L., Bianco A.C., Jonklaas J., Peeters R.P. (2017). Hypothyroidism. Lancet.

[10] Martin S.S., Daya N., Lutsey P.L., Matsushita K., Fretz A., McEvoy J.W., Blumenthal R.S., Coresh J., Greenland P., Kottgen A. (2017). Thyroid Function, Cardiovascular Risk Factors, and Incident Atherosclerotic Cardiovascular Disease: The Atherosclerosis Risk in Communities (ARIC) Study. J. Clin. Endocrinol. Metab..

[11] Bastos D.C.d.S., Chiamolera M.I., Silva R.E., de Souza M.D.C.B., Antunes R.A., Souza M.M., Mancebo A.C.A., Arêas P.C.F., Reis F.M., Turco E.G.L. (2023). Metabolomic analysis of follicular fluid from women with Hashimoto thyroiditis. Sci. Rep..

[12] Boelen A., Zwaveling-Soonawala N., Heijboer A.C., van Trotsenburg A.S.P. (2023). Neonatal screening for primary and central congenital hypothyroidism: Is it time to go Dutch?. Eur. Thyroid J..

[13] Hu F.B. (2003). Sedentary lifestyle and risk of obesity and type 2 diabetes. Lipids.

[14] LeBlanc J.G., Milani C., de Giori G.S., Sesma F., van Sinderen D., Ventura M. (2013). Bacteria as vitamin suppliers to their host: A gut microbiota perspective. Curr. Opin. Biotechnol..

[15] Natividad J.M., Verdu E.F. (2013). Modulation of intestinal barrier by intestinal microbiota: Pathological and therapeutic implications. Pharmacol. Res..

[16] Sekirov I., Russell S.L., Antunes L.C., Finlay B.B. (2010). Gut microbiota in health and disease. Physiol. Rev..

[17] Fenneman A.C., Bruinstroop E., Nieuwdorp M., van der Spek A.H., Boelen A. (2023). A Comprehensive Review of Thyroid Hormone Metabolism in the Gut and Its Clinical Implications. Thyroid.

[18] Knezevic J., Starchl C., Tmava Berisha A., Amrein K. (2020). Thyroid-Gut-Axis: How Does the Microbiota Influence Thyroid Function?. Nutrients.

[19] Li Y., Teng D., Ba J., Chen B., Du J., He L., Lai X., Teng X., Shi X., Li Y. (2020). Efficacy and Safety of Long-Term Universal Salt Iodization on Thyroid Disorders: Epidemiological Evidence from 31 Provinces of Mainland China. Thyroid.

[20] Segata N., Izard J., Waldron L., Gevers D., Miropolsky L., Garrett W.S., Huttenhower C. (2011). Metagenomic biomarker discovery and explanation. Genome. Biol..

[21] Jiang W., Lu G., Qiao T., Yu X., Luo Q., Tong J., Fan S., Chai L., Gao D., Wang R. (2023). Integrated microbiome and metabolome analysis reveals a distinct microbial and metabolic signature in Graves’ disease and hypothyroidism. Heliyon.

[22] Smirnov V.E., Makhovskiĭ V.Z. (1984). Cicatricial stenosis of the stomach and small intestine after a chemical burn. Khirurgiia.

[23] Singh N., Gurav A., Sivaprakasam S., Brady E., Padia R., Shi H., Thangaraju M., Prasad P.D., Manicassamy S., Munn D.H. (2014). Activation of Gpr109a, receptor for niacin and the commensal metabolite butyrate, suppresses colonic inflammation and carcinogenesis. Immunity.

[24] Ren Z., Zhao Z., Wang Y., Huang K. (2011). Preparation of selenium/zinc-enriched probiotics and their effect on blood selenium and zinc concentrations, antioxidant capacities, and intestinal microflora in canine. Biol. Trace Elem. Res..

[25] Fröhlich E., Wahl R. (2019). Microbiota and Thyroid Interaction in Health and Disease. Trends. Endocrinol. Metab..

[26] Xie L., Zhao H., Chen W. (2023). Relationship between gut microbiota and thyroid function: A two-sample Mendelian randomization study. Front. Endocrinol..

[27] Ishaq H.M., Mohammad I.S., Guo H., Shahzad M., Hou Y.J., Ma C., Naseem Z., Wu X., Shi P., Xu J. (2017). Molecular estimation of alteration in intestinal microbial composition in Hashimoto’s thyroiditis patients. Biomed. Pharmacother..

[28] Jiang W., Yu X., Kosik R.O., Song Y., Qiao T., Tong J., Liu S., Fan S., Luo Q., Chai L. (2021). Gut Microbiota May Play a Significant Role in the Pathogenesis of Graves’ Disease. Thyroid.

[29] Mendoza-León M.J., Mangalam A.K., Regaldiz A., González-Madrid E., Rangel-Ramírez M.A., Álvarez-Mardonez O., Vallejos O.P., Méndez C., Bueno S.M., Melo-González F. (2023). Gut microbiota short-chain fatty acids and their impact on the host thyroid function and diseases. Front. Endocrinol..

[30] Vought R.L., Brown F.A., Sibinovic K.H., McDaniel E.G. (1972). Effect of changing intestinal bacterial flora on thyroid function in the rat. Horm. Metab. Res..

[31] Nicola J.P., Nazar M., Mascanfroni I.D., Pellizas C.G., Masini-Repiso A.M. (2010). NF-kappaB p65 subunit mediates lipopolysaccharide-induced Na(+)/I(-) symporter gene expression by involving functional interaction with the paired domain transcription factor Pax8. Mol. Endocrinol..

[32] VélEz M.L., Costamagna E., Kimura E.T., Fozzatti L., Pellizas C.G., Montesinos M.M., Lucero A.M., Coleoni A.H., Santisteban P., Masini-Repiso A.M. (2006). Bacterial lipopolysaccharide stimulates the thyrotropin-dependent thyroglobulin gene expression at the transcriptional level by involving the transcription factors thyroid transcription factor-1 and paired box domain transcription factor 8. Endocrinology.

[33] Nicola J.P., Vélez M.L., Lucero A.M., Fozzatti L., Pellizas C.G., Masini-Repiso A.M. (2009). Functional toll-like receptor 4 conferring lipopolysaccharide responsiveness is expressed in thyroid cells. Endocrinology.

