# Supplementary material for: Metagenomic Analysis of Gut Microbiota Structure and Function in Adults with Subclinical Hypothyroidism: A Cross-Sectional Study in China
Source: Microorganisms. 2025 Nov 20;13(11):2643. doi: 10.3390/microorganisms13112643 (PMC12654992; doi:10.3390/microorganisms13112643)
Supplement: Supplementary file 1 [file microorganisms-13-02643-s001.zip › legends.pdf]

Figure S1. Differential functional analysis of the gut microbiota based on KEGG. (A) Differential functions in KEGG level 2 between OH and euthyroidism groups; (B) Differential functions in KEGG level 3 between OH and euthyroidism groups. The Wilcoxon rank-sum test was used to detect significant changes. *, $p < 0.05$; **, $p < 0.01$.

Figure S2. Comparison of alpha and beta diversity of gut microbiota between euthyroidism (n=222) and SCH (n=50) groups. (A) Alpha diversity analysis assessed by the Ace, Shannon, Simpson, Sobs, and Chao indices. The center line in the boxplots represents the median and the box limits indicate the interquartile range (IQR). (B) Principal component (PCoA) analysis based on Bray-Curtis distance. Permutational multivariate ANOVA (PERMANOVA) was used to examine the significance of differences in microbial community structure between groups (R^2 =0.01, p =0.002). X- and Y-axes represent the first PCoA1 and the second PCoA2, respectively. The percentage in the brackets represents the relative contribution of the component to the total difference. Each sample corresponded to one dot in the graph. Different groups are represented by different colors. (C) Differences in species-level similarity based on Bray-Curtis distance between samples across different groups were assessed using the Willcoxon test. ***, p <0.001.

Figure S3. The differential microbiota at various taxonomic levels between two groups was determined by linear discriminant analysis (LDA) and effect size (LEfSe) analysis (LDA value >3, p <0.05). (A) LEfSe taxonomic cladogram. (B) Histogram of the LDA scores. The letter in the former of the name of bacteria indicates different taxa levels. g, indicates genus; f, indicates family; o, indicates order; c, indicates class; p, indicates phylum.
