# Supplementary material for: Metagenomic Analysis of Gut Microbiota Structure and Function in Adults with Subclinical Hypothyroidism: A Cross-Sectional Study in China
Source: Microorganisms. 2025 Nov 20;13(11):2643. doi: 10.3390/microorganisms13112643 (PMC12654992; doi:10.3390/microorganisms13112643)
Supplement: Supplementary file 1 [file microorganisms-13-02643-s001.zip › supplemental methods.pdf]

Supplemental Methods

Shotgun Metagenomic Sequencing

1. Processing of metagenome sequencing data.

The data were analyzed on the free online platform of Majorbio Cloud Platform (www.majorbio.com). Briefly, the raw sequencing reads were trimmed of adapters, and low-quality reads (length <50 bp or with average quality value <20) were removed by fastp (https://github.com/OpenGene/fastp, version 0.20.0). After quality control, the number of clean reads per sample ranged from 78,211,730 to 186,041,080, with clean reads representing over 98% of the raw reads for all samples, indicating high data quality suitable for downstream analyses. Reads were aligned to the human genome by BWA (http://bio-bwa.sourceforge.net, version 0.7.17) and any hit associated with the reads and their mated reads were removed.

The quality-filtered data were assembled using MEGAHIT (https://github. com/voutcn/megahit, version 1.1.2). Contigs with a length ≥300 bp were selected as the final assembling result. Open reading frames (ORFs) from each assembled contigs were predicted using Prodigal（https://github.com/hyattpd/Prodigal, version 2.6.3）and a length ≥100 bp ORFs were retrieved.

A non-redundant gene catalog was constructed using CD-HIT (http://weizhongli-lab.org/cd-hit/, version 4.7) with 90% sequence identity and 90% coverage. Gene abundance for a certain sample was eatimated by SOAPaligner (https://github.com/ShujiaHuang/SOAPaligner，version soap 2.21 release) with 95% identity.

2. Taxonomic and functional annotation.

The best-hit taxonomy of non-redundant genes was obtained by aligning them against the NCBI NR database (20230830) by DIAMOND (http://ab.inf.uni-tuebingen.de/software/diamond/, version 2.0.13) with an e-value cutoff of $1e^{-5}$. Similarly, the functional annotation (KEGG) of non-redundant genes was obtained. Differential analyses across taxonomic, functional, and gene-wise levels were performed based on annotated profiles and gene abundance data, using the Kruskal-

Wallis test, LEfSe analysis, and the Wilcoxon rank-sum test as needed.
