# Supplementary figures and images for: Metagenomic Analysis of Gut Microbiota Structure and Function in Adults with Subclinical Hypothyroidism: A Cross-Sectional Study in China
Source: Microorganisms. 2025 Nov 20;13(11):2643. doi: 10.3390/microorganisms13112643 (PMC12654992; doi:10.3390/microorganisms13112643)

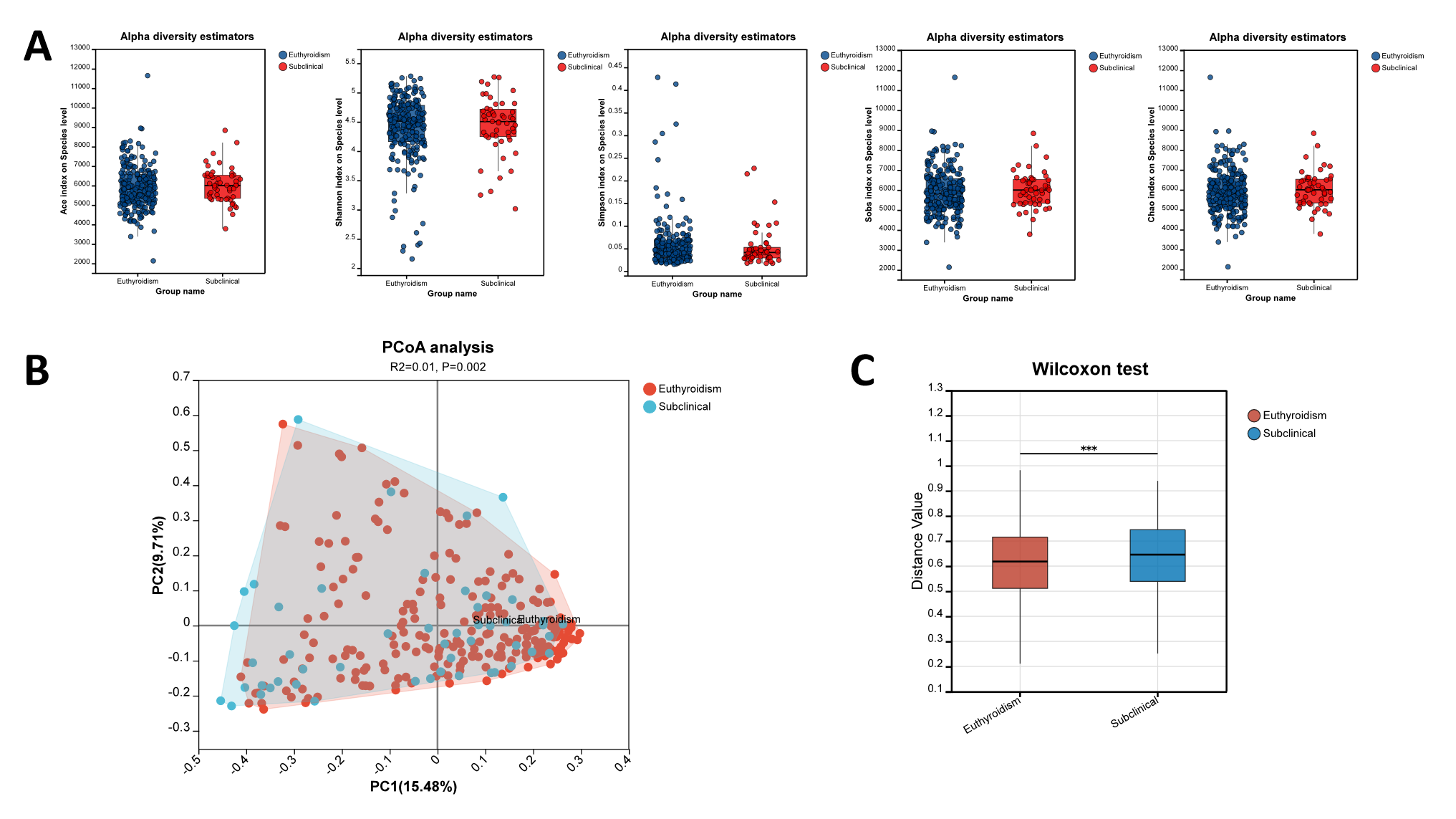

Supplement: Supplementary file 1 [file microorganisms-13-02643-s001.zip › Figure S2.tif]

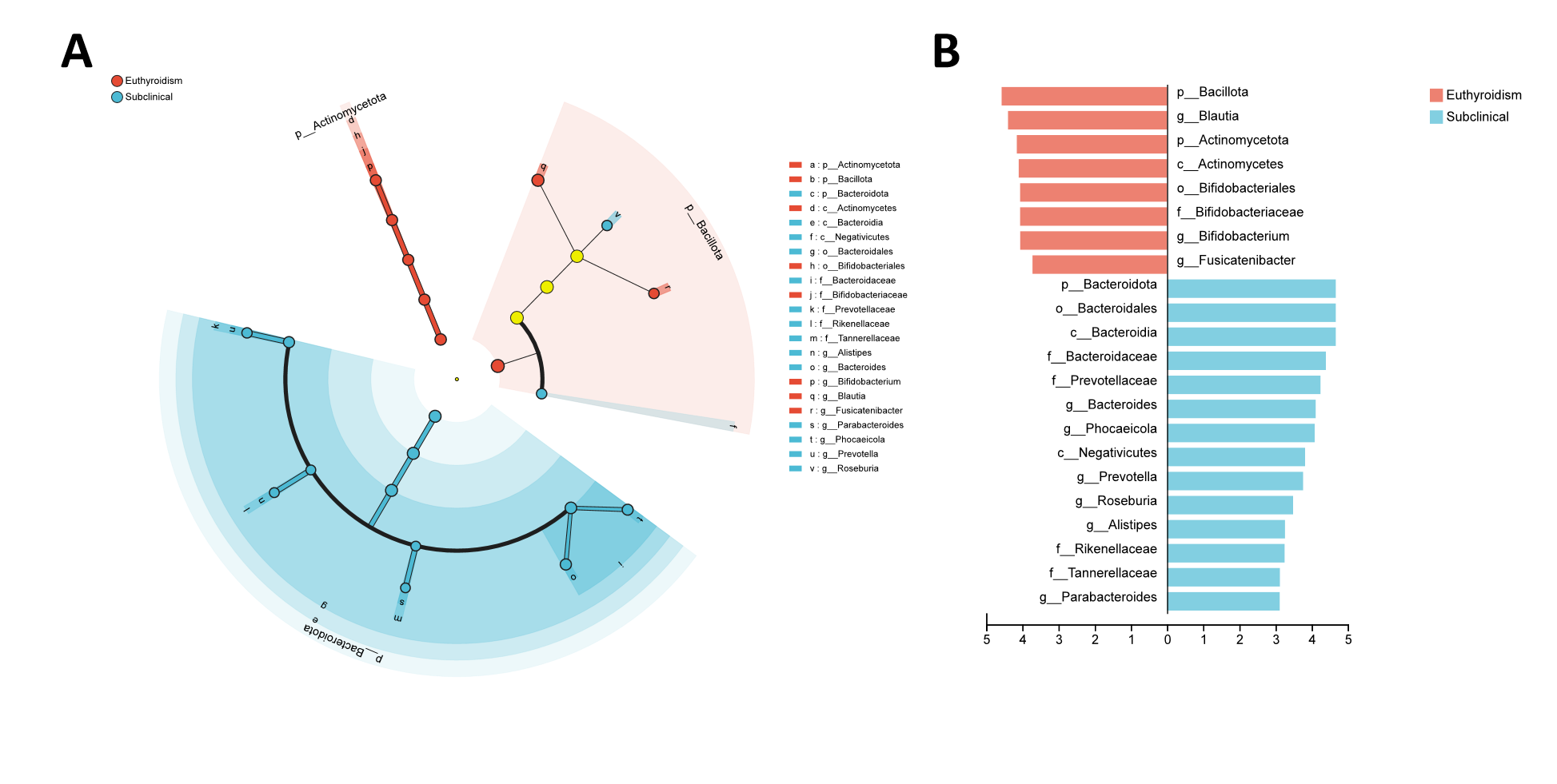

Supplement: Supplementary file 1 [file microorganisms-13-02643-s001.zip › Figure S3.tif]

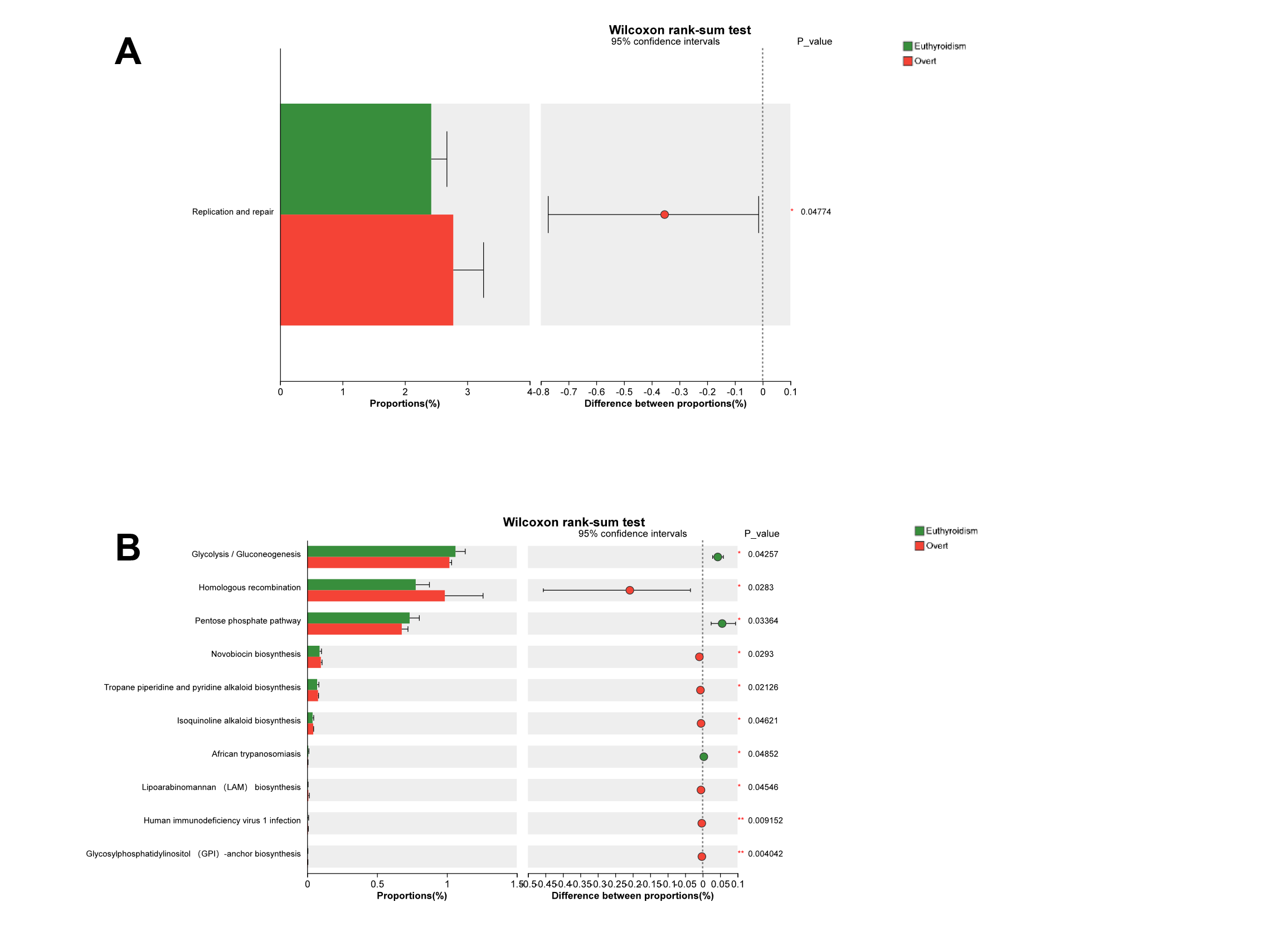

Supplement: Supplementary file 1 [file microorganisms-13-02643-s001.zip › Figure S1.tif]
